# Supraventricular tachycardia and atrial flutter associated with a coronary sinus diverticulum: A case report

**DOI:** 10.3892/etm.2013.1050

**Published:** 2013-04-05

**Authors:** XIAOLIN WU, RUI ZHU, HONG JIANG, WENWEI LIU

**Affiliations:** 1 Department of Cardiology, Renmin Hospital of Wuhan University, Wuhan, Hubei 430060;; 2 Department of Cardiology, Xiangyang Central Hospital, Affiliated Hospital of Hubei University of Arts and Science, Xiangyang, Hubei 441000, P.R. China

**Keywords:** supraventricular tachycardia, atrial flutter, coronary sinus diverticulum, catheter ablation

## Abstract

The case of a patient with narrow QRS-complex supraventricular tachycardia and atrial flutter is described. The 12-lead surface electrocardiogram (ECG) revealed sinus rhythm with ventricular pre-excitation and negative δ waves in leads II, III and aVF, indicating Wolff-Parkinson-White syndrome with a posteroseptal accessory pathway (AP). Coronary sinus angiography revealed the presence of a diverticulum near the coronary sinus ostium. The AP was successfully ablated using radiofrequency energy applied in the neck of the diverticulum, following several failed attempts at catheter ablation from the endocardial surface of the posteroseptal space.

## Introduction

Radiofrequency catheter ablation (RFCA) has become the curative treatment of choice for supraventricular tachycardia (SVT) in patients with Wolff-Parkinson-White (WPW) syndrome (1). The posteroseptal accessory pathways (APs) are occasionally located in the epicardial region and are associated with ablation failure due to their complex anatomic arrangement. This type of epicardial AP results from a connection between an extension of the coronary sinus (CS) myocardial coat along the middle cardiac vein, or the neck of a CS diverticulum and the left ventricular epicardium (2). In the current study, a case of left posteroseptal AP showing narrow QRS-complex SVT and atrial flutter is described that was successfully ablated with radiofrequency energy applied in the neck of a CS diverticulum.

## Case report

**Figure 1:**
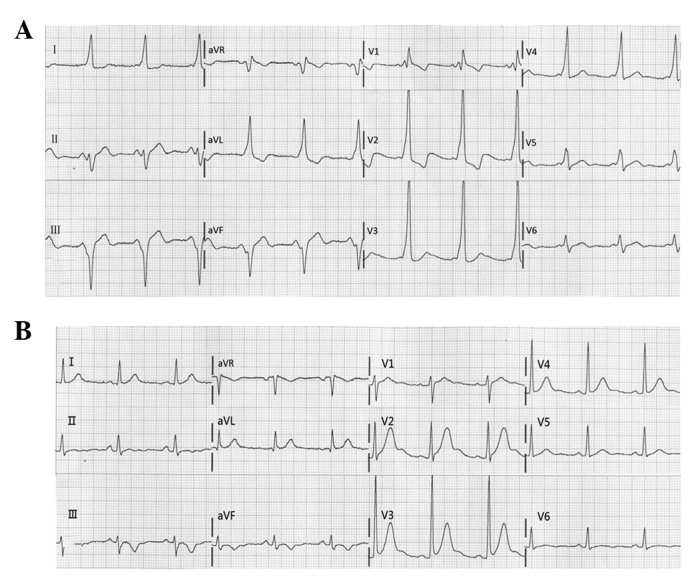
Twelve-lead surface electrocardiograms (ECGs) showing (A) pre-excitation and (B) the disappearance of pre-excitation following radiofrequency catheter ablation.

**Figure 2:**
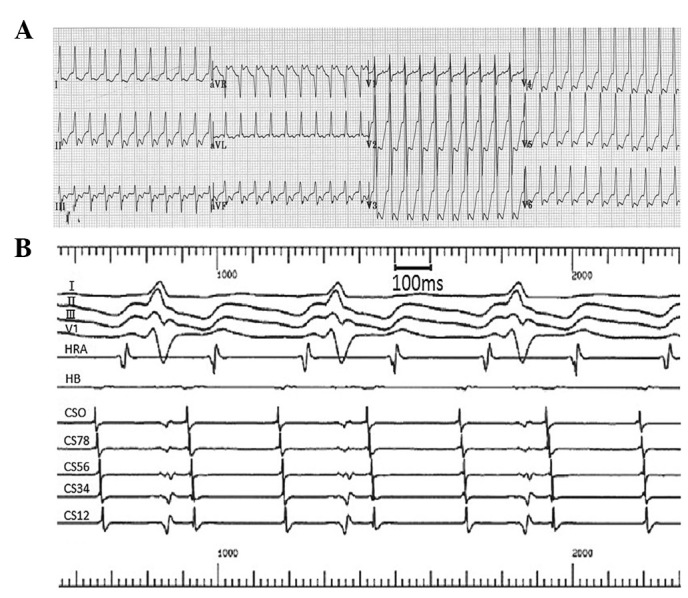
(A) Electrocardiogram (ECG) showing SVT during the attacks. (B) Intracardiac electrogram showing paroxysmal atrial flutter (2:1) during ablation.

A 46-year-old male presented with recurrent SVT associated with symptoms of palpitations, dizziness and diaphoresis. The termination of tachycardia with drugs is not always successful and electrical cardioversion may be required. The patient in this study used antiarrhythmic drugs prior to radio-frequency catheter ablation (RFCA) and drugs alone were used during the attacks. A sinus rhythm electrocardiogram (ECG) showed ventricular pre-excitation with a positive δ wave in leads I, V1, V2 and a negative δ wave in leads II, III and aVF (Fig. 1A), indicating a left posteroseptal AP. SVT at a rate of 230 beats/min was documented by ECG during the attacks (Fig. 2A). The rhythm was regular and the waves showed a narrow QRS-complex. The patient also presented with paroxysmal atrial flutter (2:1) during ablation (Fig. 2B). The esophagus electrophysiological study (EPS) confirmed the manifest left AP accompanied orthodromic atrioventricular reentrant tachycardia (AVRT). Clinical examination, chest X-ray and echocardiography were normal.

**Figure 3:**
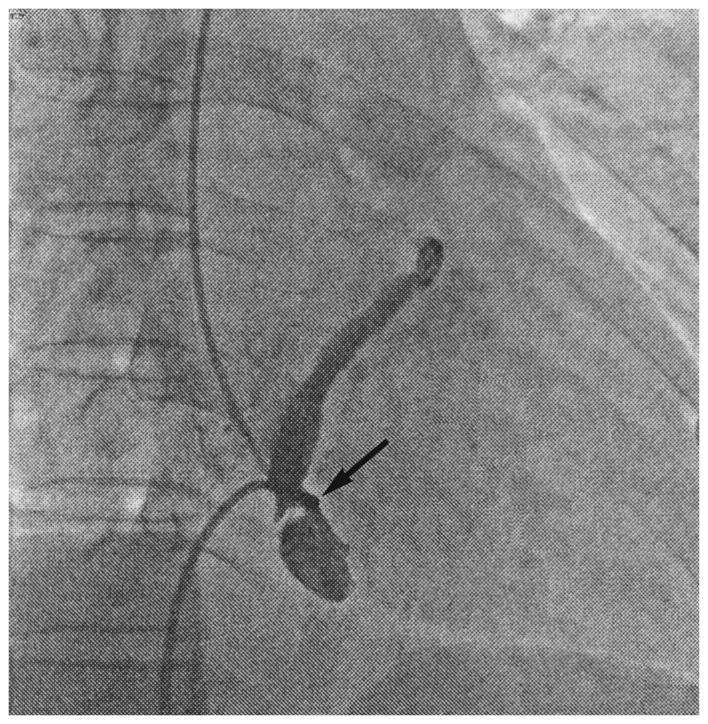
Coronary sinus angiography (anteroposterior) showing a diverticulum with a narrow neck (arrow) near the ostium.

**Figure 4:**
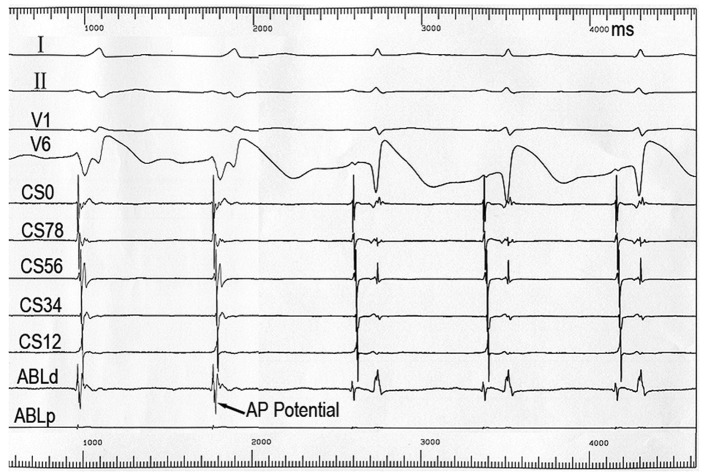
Recording from the neck of diverticulum during sinus rhythm. The earliest atrial activation is seen in CS0, which is just within the coronary sinus ostium. The accessory pathway (AP) potential is recorded by the ablation catheter. The AV wave separated and the AP potential disappeared. CS, coronary sinus; ABL, ablation

An EPS was performed to identify the mechanism of tachycardia and RFCA. A deflectable decapolar catheter (Cordis Webster, Diamond Bar, CA, USA) was placed in the coronary sinus via the left subclavian vein. Quadripolar catheters (Cordis-Webster) were introduced through the right femoral vein and positioned in the His-bundle region and the right ventricular apex (RVA). The intracardiac electrogram revealed the earliest atrial activation was at the CS ostium and RVA pacing initiated orthodromic AVRT. The AP was located in the left posteroseptal region. An 8-French ablation catheter with a 4-mm tip electrode (Cordis-Webster) was introduced via the right femoral artery and placed under the mitral valve close to the annulus. The ventricular wave could not be significantly ahead of the atrial wave and the AV wave was not always merged. The applications of radiofrequency energy (20W, 10 sec) delivered in the posteroseptal region of the mitral valve annulus failed to ablate the AP. Due to the consideration that the AP may have been located at the epicardium, CS angiography was implemented and a CS diverticulum was identified near the ostium (Fig. 3). The ablation catheter was placed into the CS via the right femoral vein and the ideal target in the neck of CS diverticulum was recorded. The ventricular wave was significantly ahead of the atrial wave and the AV wave was fusion well (Fig. 4). At this site, a clear AP potential was recorded by the ablation catheter. Ablation in this area under temperature control with a radiofrequency energy of 20 W (AV wave separation after 3 sec then terminated discharge after 60 sec, Fig. 4) achieved successful abolition of atrioventricular conduction via the AP. Following ablation, the δ wave disappeared from the surface ECG and the PR interval was 0.16 sec (Fig. 1B). Programmed atrial pacing showed the retrograde atrioventricular conduction property and ventricular pacing displayed AV wave separation. A vigorous stimulation protocol with isoprenaline failed to induce tachycardia. The patient did not experience recurring tachycardia within the one-year follow-up.

## Discussion

A CS diverticulum is a rare congenital abnormality of the intracardiac veins and is shown to contain myocardial fibers which connect to the ventricle and the CS myocardial coat. The connections between the CS myocardial coat and the ventricle may serve as an AP (2,3). CS diverticula are known to be present in patients with WPW syndrome with APs located in the posteroseptal region (2,3). Epicardial APs are most commonly located in the posteroseptal regions. The finding of a steep negative δ wave in lead II is known to be predictive of an epicardial AP. It has been reported that the sensitivity of a negative δ wave in lead II in identifying a CS AP is >70% (4). The ECG of the present case showed a positive δ wave in leads I, V1, V2 and negative δ waves in leads II, III and aVF. The patient was confirmed as having left posteroseptal epicardial AP accompanied by a CS diverticulum.

A CS diverticulum is usually associated with manifest posteroseptal APs and the manifest APs have bidirectional atrioventricular conduction properties (5). These APs are difficult to ablate endocardially outside the diverticulum and successful ablation is often performed from within the diverticulum. Diagnosis of CS diverticulum may be difficult from transthoracic echocardiograms. CS angiography is suggested, therefore, prior to catheter ablation of posteroseptal APs, especially when endocardial signals are not optimal. In patients with a CS diverticulum, an AP potential is often recorded from the neck of the diverticulum. In the EPS, orthodromic AVRT was easily inducible and the earliest atrial activation during sinus rhythm was mapped and identified to be within the CS diverticulum. A CS diverticulum may contribute to the reentrant circuit of atrial flutter (6). The present patient presented with paroxysmal atrial flutter (2:1) during surgery. WPW syndrome with a CS diverticulum has a high incidence of atrial flutter with short R-to-R intervals, and a number of sudden mortalities have been reported. APs in this location tend to have a very rapid atrioventricular conduction, putting these patients at risk of sudden mortality during atrial flutter or atrial fibrillation; such APs, therefore, should be ablated (7,8). Due to the close proximity of the CS ostium and posterolateral branch of the right coronary artery, caution should be exercised while applying radiofrequency energy to the CS. When the optimal ablation site is near the passage of the right coronary artery, saline-irrigated ablation or temperature-controlled ablation is recommended to avoid the complication of coronary artery stenosis (9). Moreover, the walls of the coronary veins and the atriums are rather thin so vessel rupture, hemopericardium and cardiac tamponade may occur when radiofrequency is delivered within a CS diverticulum. CS angiography was performed prior to the ablation to define the anatomy around the ablation site and a temperature-controlled ablation catheter was used to prevent complications. It is recognized that ablation of a posteroseptal AP requires CS angiography and careful evaluation of CS recordings for AP potential.

This case report illustrates a patient who had SVT and atrial flutter with a left posteroseptal AP associated with a CS diverticulum. RFCA at the neck of the CS diverticulum effectively interrupted the AP conduction. The current case highlights the potential importance of CS angiography and identification of AP potentials in patients with a posteroseptal AP which is difficult to ablate by the endocardial approach.
